# Inverted T‐Shape Connective Tissue Graft for Interdental Papilla Reconstruction: A Clinical Case Series

**DOI:** 10.1111/jerd.70035

**Published:** 2025-09-25

**Authors:** Abdusalam E. Alrmali, Vanessa Frazao Cury, Jessica Latimer, Preston D. Miller, Hom‐Lay Wang

**Affiliations:** ^1^ Department of Periodontics and Oral Medicine University of Michigan School of Dentistry Ann Arbor Michigan USA; ^2^ Department of Oral Pathology, Oral Medicine and Oral and Maxillofacial Surgery University of Tripoli, School of Dentistry Tripoli Libya; ^3^ Private Practice Belo Horizonte Brazil; ^4^ Department of Oral Medicine, Infection, and Immunity Harvard School of Dental Medicine Boston Massachusetts USA; ^5^ Department of Periodontics Medical University of South Carolina College of Dental Medicine Charleston South Carolina USA

**Keywords:** case study, connective tissue, esthetics‐dental, gingival recession

## Abstract

**Objective:**

To evaluate the clinical application and outcomes of the inverted T‐shape connective tissue graft (IT‐CTG) technique for reconstructing deficient interdental papillae in cases of advanced gingival recession, focusing on both quantitative and qualitative results in a consecutive case series.

**Clinical Considerations:**

The IT‐CTG technique was performed using a remotely mucoperiosteal tunnel approach, with the donor graft harvested in an inverted T configuration from the palate. Patient selection included five individuals with RT2 or RT3 recession and papilla loss in the esthetic zone, following defined inclusion and exclusion criteria. All surgical steps were standardized, including graft shape, the use of thin suture material, and a consistent postoperative care protocol. Quantitative outcomes such as papilla height gain and Jemt's papilla index improvement were assessed at 3, 6, and 12 months. Among five treated sites, complete papilla fill (Jemt index score 2) was achieved in two sites (both RT3 cases; 40%) after restorative procedures had been performed, and partial fill (Jemt index score 1–2, corresponding to 50%–75% improvement) was observed in the remaining three sites (RT2 cases; 60%). The mean Jemt's papilla index improved from a baseline of 0.2 to 1.8 in the final follow‐up. Acceptable esthetic outcomes and minimal morbidity were consistently noted across all patients. The addition of restorative procedures is expected to further enhance papilla fill and support creeping attachment over time.

**Conclusions:**

The inverted T‐shape connective tissue graft technique achieved acceptable papilla reconstruction, satisfactory esthetic outcomes, and minimal complications in this case series. While the results are promising, further controlled studies with larger sample sizes and longer follow‐up are necessary to confirm the efficacy and broader applicability of this approach.

**Clinical Significance:**

Loss of interdental papilla in advanced gingival recession presents a significant esthetic and functional challenge, often resulting in compromised smile esthetics and patient dissatisfaction. The IT‐CTG technique offers an innovative surgical solution for interdental papilla reconstruction, especially in cases where traditional approaches may be limited by poor vascularity and difficult access. By employing enhanced vascularization and a remotely tunneled approach, IT‐CTG may improve esthetic outcomes and restore papillary architecture in the anterior esthetic zone. This technique is particularly beneficial in cases of RT2 and RT3 recession with papilla loss, where achieving optimal fill in a single procedure is challenging. Integration of IT‐CTG into clinical practice expands the treatment options for clinicians managing complex mucogingival defects, though careful case selection and adjunctive restorative procedures may be required to support complete papilla regeneration and long‐term stability.

## 1 Introduction

Recession type 2 and 3 (RT2 and RT3) defects pose significant challenges in mucogingival surgery, particularly in cases involving the reconstruction of deficient interdental papillae (IP) [1, 2]. The loss of IP is directly correlated with the loss of interproximal bone, which further limits the already compromised blood supply in this region [1, 2, 3]. This reduced vascularity, combined with the narrow dimensions of interproximal tissue, makes IP reconstruction one of the most difficult and least predictable procedures in periodontal plastic surgery. The interproximal area is inherently challenging due to its anatomical constraints, including minimal circulation and limited access for surgical manipulation [4].

In 1992, Tarnow and colleagues described the critical dimensions of the interproximal space between natural teeth, outlining expectations regarding papilla fill. According to their findings, papilla fill is likely to occur when the vertical distance between the interdental contact point and the interproximal bone is less than 5 mm [5]. However, this rule may not be universally applicable, as individual anatomical variations and specific patient factors can influence such parameters. Factors such as the presence of interproximal bone loss, the shape of the interdental contact point, and the overall health of the surrounding gingiva may affect the predictability of papilla fill [6].

Several surgical techniques have been proposed to increase papilla volume through soft tissue grafting, which share similar principles with mucogingival defect correction [7, 8, 9]. These techniques demonstrate adequate evidence supporting predictable treatment of gingival recession type 1 (RT1) defects, but complete reconstruction of lost IP remains challenging, particularly in cases with severe periodontal disease and interproximal bone loss [1]. Recent studies have explored innovative approaches, including hyaluronic acid and platelet‐rich fibrin (PRF) for enhancing papilla reconstruction, though results vary; this outcome may relate to I‐PRF influencing gingival thickness more than papilla height [10, 11]. The tunnel subepithelial connective tissue graft technique, which maintains flap vascularization, also offers predictable outcomes and promotes early healing [12, 13].

Reconstructing lost interdental papillae in esthetically demanding areas remains one of the key objectives for periodontists. Both non‐surgical and surgical approaches have been developed to address soft tissue deformities in interproximal tissues, including prosthetic coverings, periodontal surgeries, orthodontic tooth alignment, or a combination of these techniques [14, 15, 16, 17, 18]. Surgical techniques targeting the “black triangle problem” related to open embrasure spaces frequently utilize free epithelialized gingival grafts, connective tissue grafts from the maxillary tuberosity, or buccally directed displacement of interproximal palatal tissue. However, these procedures have shown limited success, primarily due to inadequate graft blood supply [19, 20]. Recent innovations, such as the 3D tunneling technique, have been proposed to improve outcomes in gingival recession and papilla reconstruction [21]. The key distinction in newer tunneling approaches lies in the enhanced graft survival and integration achievable by optimizing blood supply, a critical factor historically limiting success—especially around implants where vascularity is already compromised [21].

Building on previous work with the inverted T‐shape free gingival graft, this report introduces a surgical technique utilizing the same foundational concept. Specifically, the application of a connective tissue graft (CTG) is explored for the management of advanced facial and interproximal defects, including interdental papilla (IP) reconstruction via a tunnel approach. The inverted T‐shape connective tissue graft (IT‐CTG) technique offers clinicians a valuable option for optimizing treatment outcomes and enhancing patient esthetic satisfaction in challenging cases of interproximal gingival recession or mucosal dehiscence. The aim of this case series is to evaluate the clinical application and outcomes of the IT‐CTG technique for reconstructing deficient interdental papillae in cases of advanced gingival recession.

## 2 Materials and Methods

This retrospective, multicenter case series was conducted in accordance with the ethical principles outlined in the Declaration of Helsinki and prepared following the PROCESS guidelines. Consecutive cases were included to evaluate the clinical application of an inverted T‐shaped connective tissue graft for the management of RT2 and RT3 gingival recession defects.

### 2.1 Preparation of the Recipient Site

**FIGURE 1:**
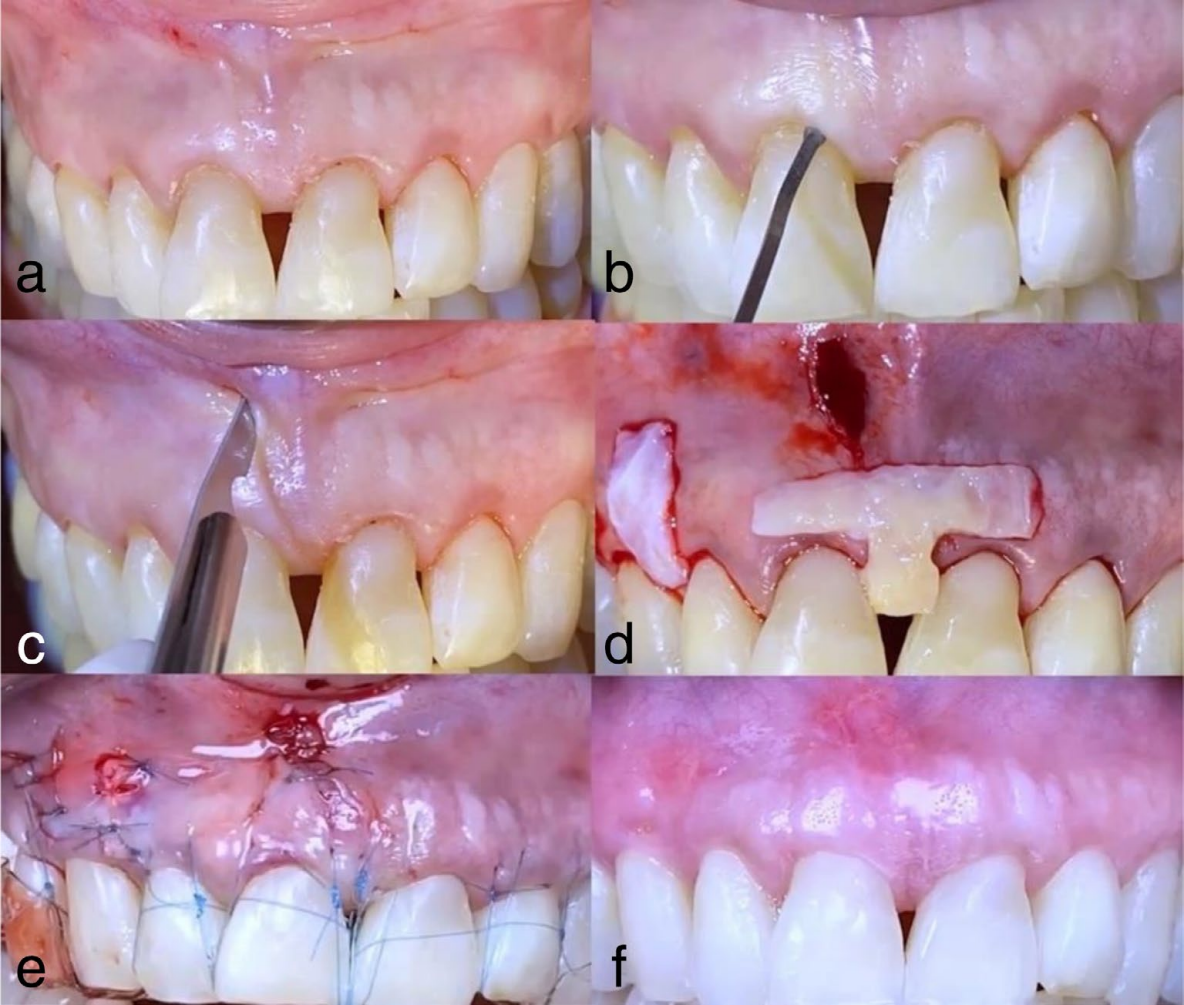
A nonsmoking, systemically healthy 66‐year‐old female. Clinical and radiographic examination revealed recession type 2 (RT2) gingival recession in her anterior maxilla, specifically at the sites of teeth #8 and #9. (a) Pre‐surgical clinical photo showing the recession defect at the facial aspect of teeth #8 and #9, with significant gingival tissue loss and root exposure. (b) Tunneling of the mesio‐buccal sulcus using a microsurgical tunneling instrument to create a donor site for the connective tissue graft (CTG) while preserving the vascular supply to the gingival tissue. (c) Vertical incision made apical to the defect site to facilitate graft placement and provide access for tissue mobilization. (d) Placement of an in situ connective tissue graft (IT‐CTG) at the defect site involving teeth #8 and #9, along with additional placement of a CTG at the #6 site to enhance the overall esthetic and functional outcome. (e) Immediately post‐surgery, showing the grafts in place with sutures securing the tissue, providing tension‐free closure. (f) Six‐month follow‐up showing successful graft integration, with complete coverage of the recession defect and maintained gingival health at the treated sites.

**FIGURE 2:**
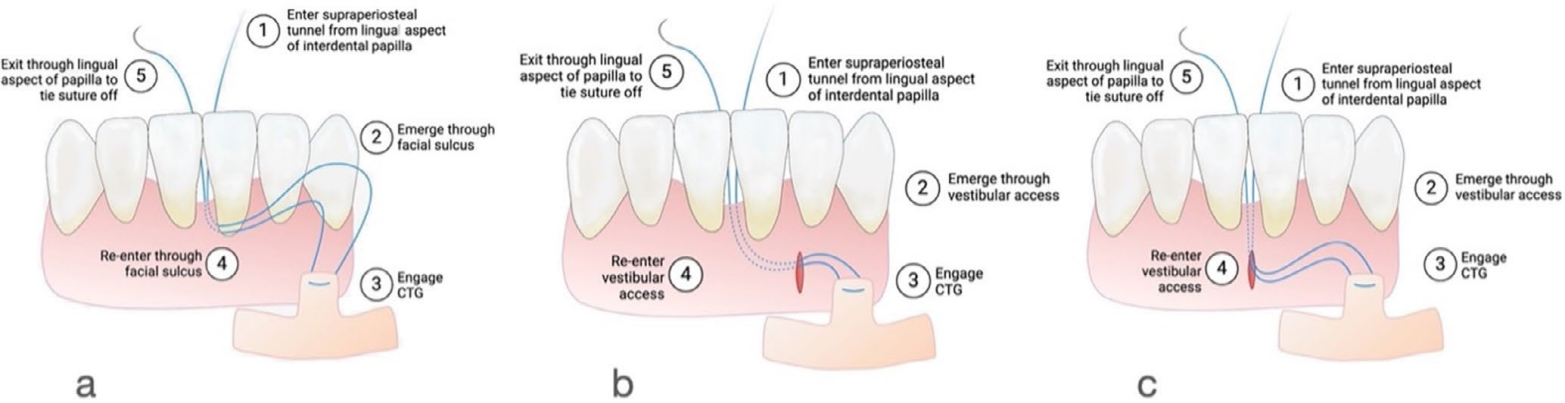
Schematic illustrations represent three different scenarios for graft insertion (a, b, c) based on the following factors: the space between the teeth, the quality of interproximal tissues, the depth of the vestibule, and the presence of frenulum attachment. (a) through the sulcus, (b) through the lateral VISTA, (c) through the frenum; care should be taken not to maintain the blood supply for the interproximal soft tissues.

**FIGURE 3:**
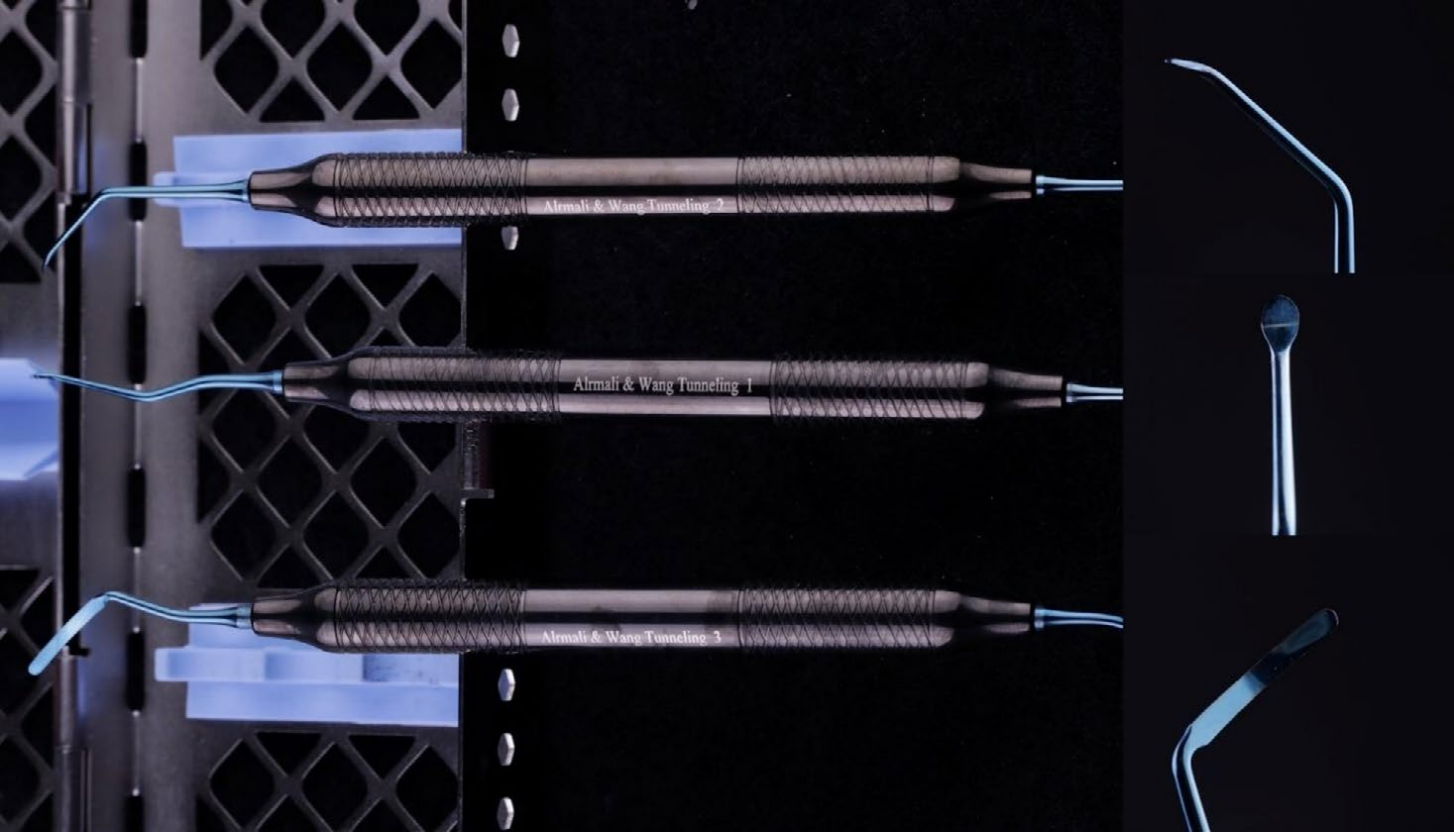
Surgical tunneling kit used for the inverted T‐shape connective tissue graft (IT‐CTG) procedure. The kit includes three specifically designed microsurgical instruments that facilitate precise access to the interdental papilla through the vestibule. These instruments enable atraumatic tunneling and optimal placement of the graft in challenging interproximal areas (Nexton, Sialkot—Pakistan).

To minimize inflammation and ensure optimal surgical conditions, an injection of lidocaine and epinephrine solution (1:100,000) was administered in the vestibule, away from the target area, and on the palate away from the prospective harvesting site. This approach avoided transpapillary and intra‐sulcular injections to prevent hypoxic conditions and physical trauma to the interdental papillae (IP), which are critical for maintaining blood supply and facilitating graft integration. Following anesthesia, straight and curved microsurgical tunneling tools were utilized to create a mucoperiosteal tunnel on the buccal side without disturbing the papilla (Figures 1 and 2). This full‐thickness tunnel was extended laterally and apically around the adjacent half of the neighboring teeth, and split thickness beyond the mucogingival junction, to minimize any possible stress on the interdental papilla (Figures 1 and 2). The use of the remote approach ensures minimal tissue trauma and preserves the natural vascularization of the recipient site, which is essential for graft survival and integration. Using a curved papilla tunneling instrument (Nexton, Sialkot—Pakistan), a 2‐mm interproximal supra‐periosteal tunnel was made under the IP to passively connect the buccal and palatal/lingual recipient sites (Figure 3). Mobility of the papilla is crucial for forming a recipient space under the papilla, positioning the connective tissue graft, and allowing for the coronal movement of the papilla.

### 2.2 Preparation of the Donor Site

**FIGURE 4:**
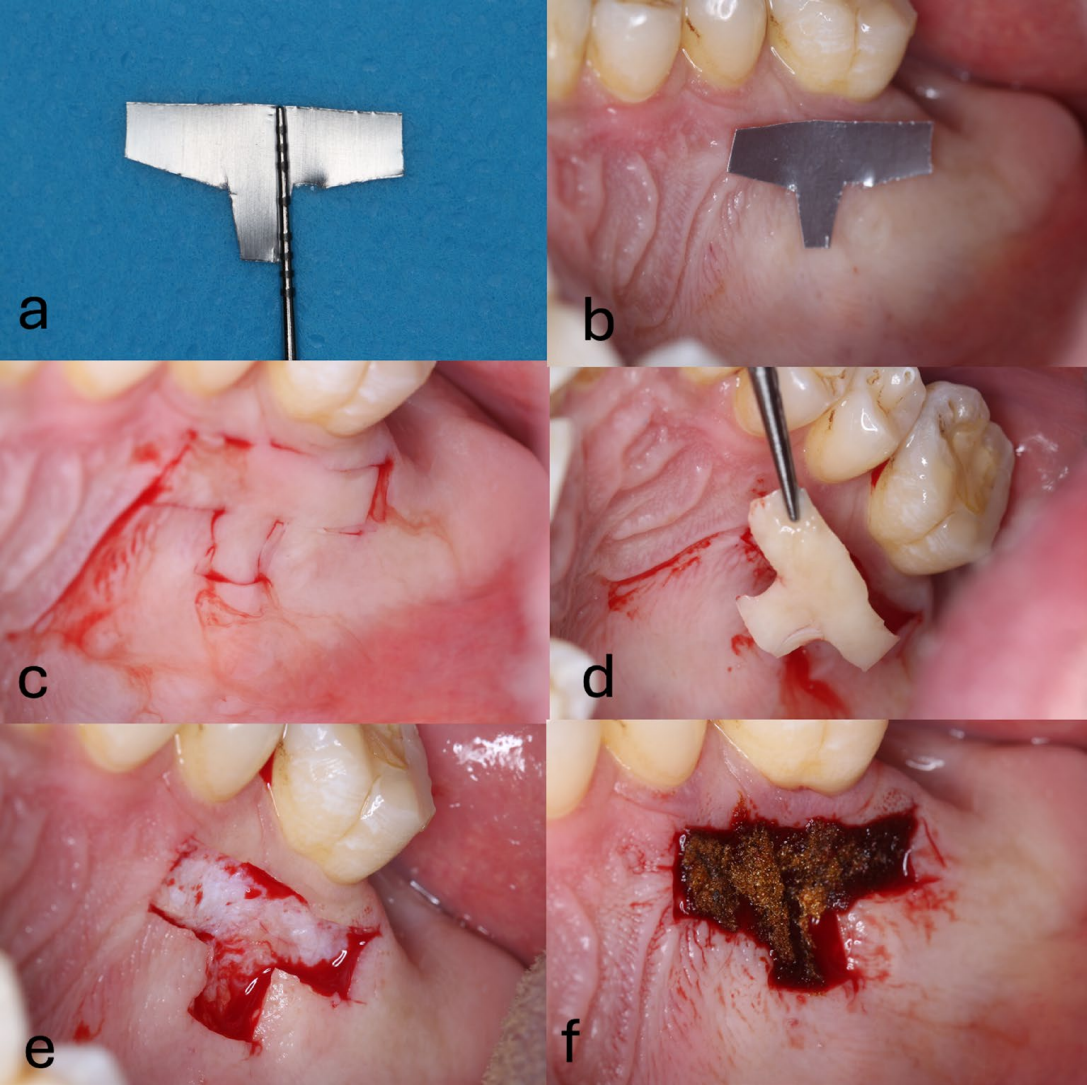
(a) Pre‐surgical clinical photo showing an inverted T‐shaped guide made from a sterile aluminum sheet. The guide is designed to outline the graft harvest site for precision and to ensure proper graft dimensions. Measurements of the T‐shaped guide illustrate the precise dimensions of the harvest area to ensure consistency and optimal graft size. (b) Photo showing the guide placed at the desired harvest site on the palate, ensuring the graft is harvested from the correct location for sufficient tissue. (c) Initial incision lines made around the guide, preparing for the removal of the free gingival graft (FGG). (d) Removal of the T‐shaped free gingival graft (FGG), prior to extra‐oral de‐epithelialization. The graft is carefully lifted to preserve its integrity and minimize trauma to the surrounding tissue. (e) Harvest site following the removal of the FGG, showing the exposed donor tissue area that will heal after the graft is removed. (f) Immediate post‐surgery photo showing collagen tape placed over the harvest site to achieve hemostasis and promote early healing.

**FIGURE 5:**
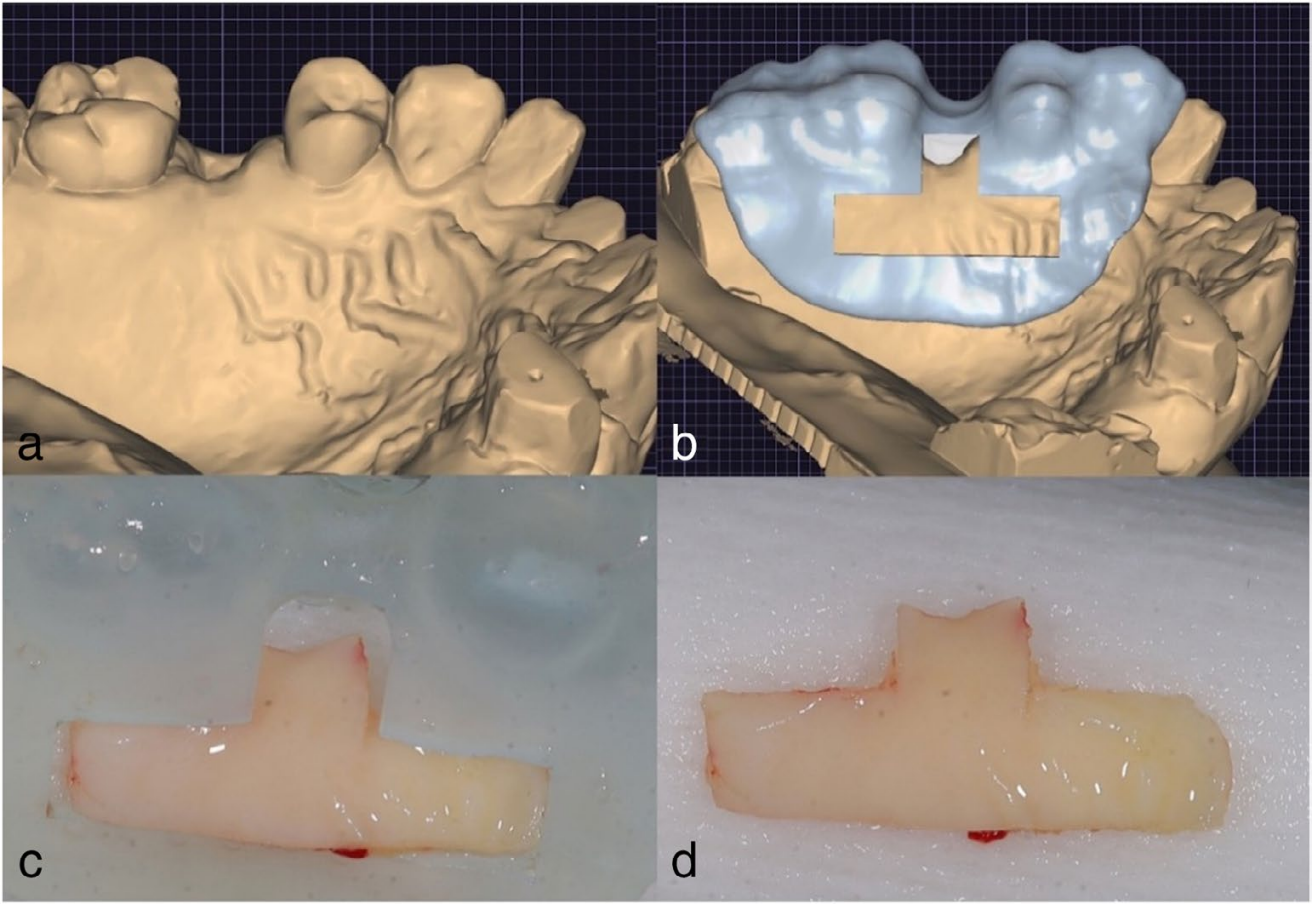
Surgical guide used for harvesting in situ connective tissue graft (IT‐CTG). (a) Digital impression of maxilla for harvest site guide fabrication. (b) Digital guide fabrication for harvest site. (c and d) Harvested FGG, before de‐epithelialization, with and without the surgical guide.

The donor site on the palate was carefully prepared using a template or surgical guide in the shape of an inverted T to ensure precise graft harvesting (Figures 4 and 5). The template was positioned 2 mm away from the adjacent gingival margin of the maxillary dentition to minimize potential damage to the surrounding tissues and ensure adequate healing of the donor site. The graft was harvested with careful attention to achieve an even thickness of around 1.5 mm, which is crucial for optimal graft integration and minimizing donor site morbidity.

**FIGURE 6:**
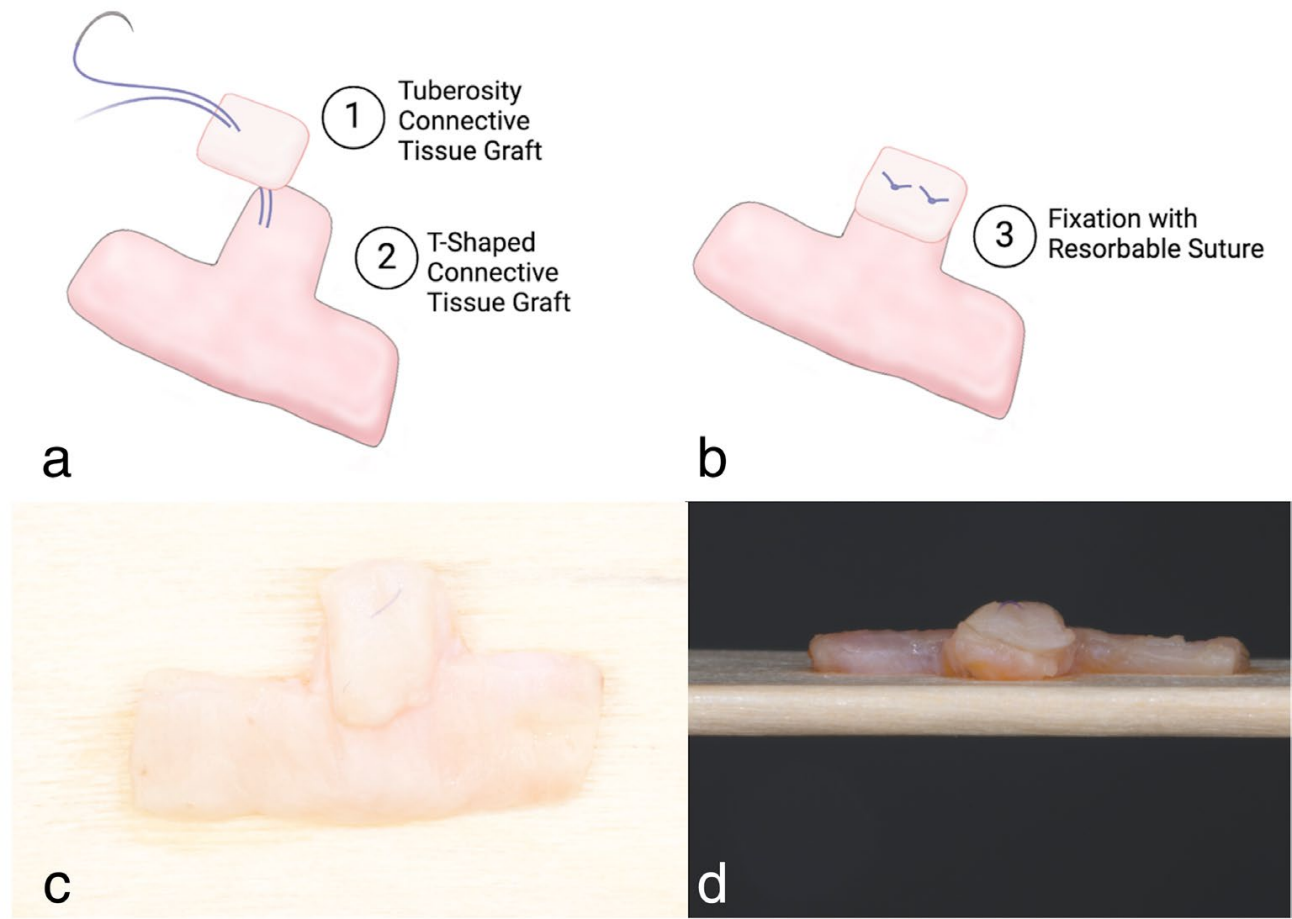
Schematic illustrations another modification involves adding a small piece from the maxillary tuberosity to the T‐CTG, which is then fixed using 7/0 polyglycolic acid with two horizontal mattress sutures. (c and d) Application of an in situ connective tissue graft (IT‐CTG) with a tuberosity graft, secured with 7‐0 PGA sutures and cross mattress suturing, in accordance with the “iceberg” CTG technique.

The final graft dimensions were approximately 10 mm in length and 4 mm in width, with a 3 mm extension for the interproximal papilla. For patients with full dentition, the 3 mm extension was directed toward the mid‐palatine raphe, maintaining a safe distance of at least 2 mm from the marginal gingiva to prevent any potential irritation or complications (Figure 4). In cases of patients with edentulous spaces, the 3 mm extension was positioned coronally toward the edentulous area, ensuring optimal graft placement and minimizing potential complications (Figure 5). Following harvesting, the graft was contoured and de‐epithelialized to enhance its integration with the recipient site. It was then inserted through the sulcus or a tunnel and secured on the lingual side of the interdental papilla using sutures (Figures 1 and 2). In one case, the IT‐CTG technique was modified to incorporate an additional tuberosity graft, which was combined with the palatal connective tissue graft (Figure 6).

### 2.3 Suture Techniques

To ensure stable positioning of the T‐shaped graft beneath the papilla, a combination of stabilizing sutures was employed. The needles were inserted under the vestibular envelope, passed through the sulcus of the adjacent tooth, re‐entered the corresponding lateral sides, and returned to their starting points. This approach helped maintain the graft in place while minimizing tissue trauma.

For the palatal suture, the needle was carefully maneuvered through the tunnel from the palatal to the buccal side, exited in the same sulcus as the previous sutures, pierced into the graft tissue, and returned to the palatal side. It is crucial that the graft's entry point is always on the buccal side, with needles exiting in the same sulcus before penetrating the corresponding flap. This technique ensures that the graft is securely positioned under the papilla without compromising its blood supply.

**FIGURE 7:**
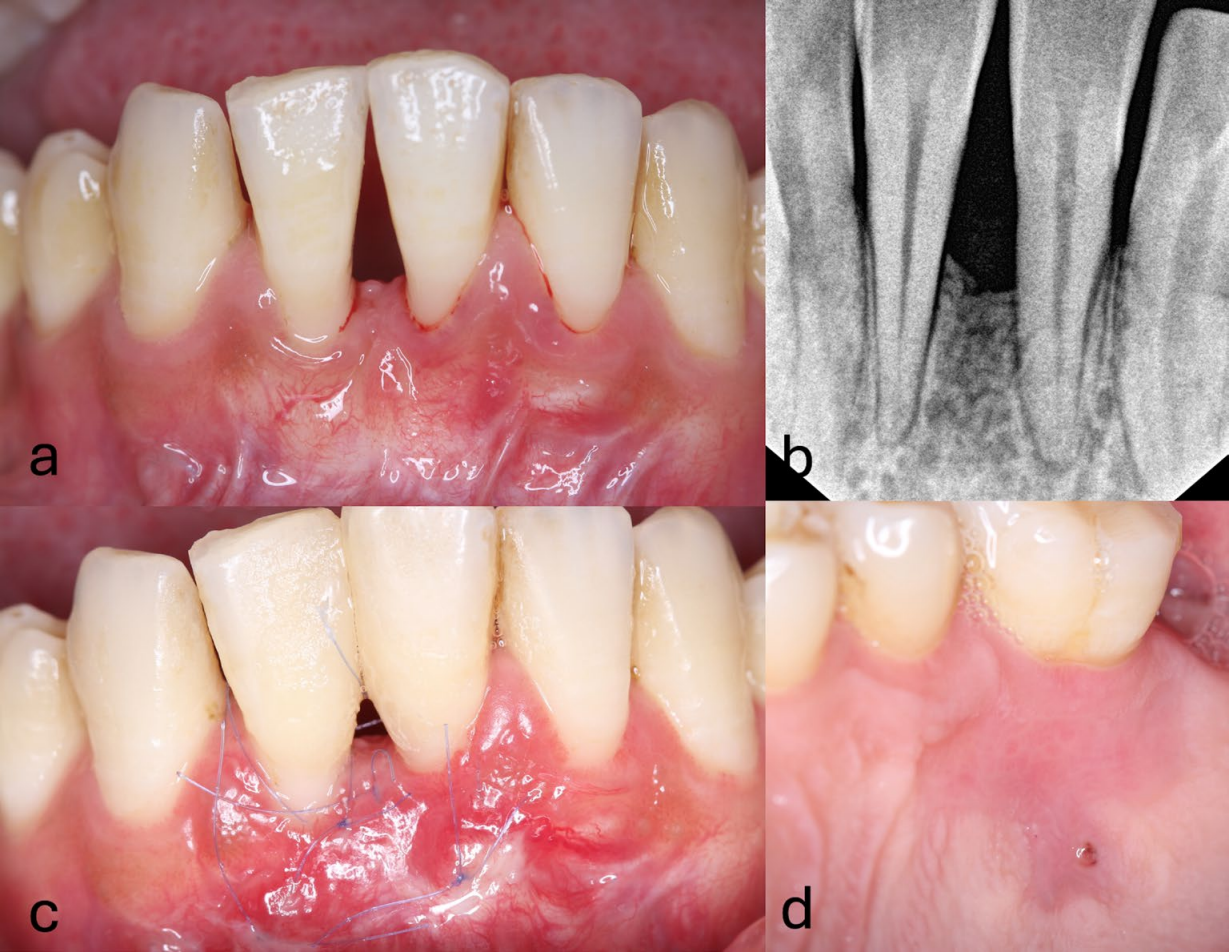
Same patient as shown in Figure 4. A nonsmoking, systemically healthy 33‐year‐old male. (a and b) demonstrating the clinical and radiographic examinations revealed recession type 3 (RT3) gingival recession in the lower anterior mandible, specifically between teeth #24 and #25. (c and d) showing 3 weeks' follow‐up from the donor and recipient sites.

**FIGURE 8:**
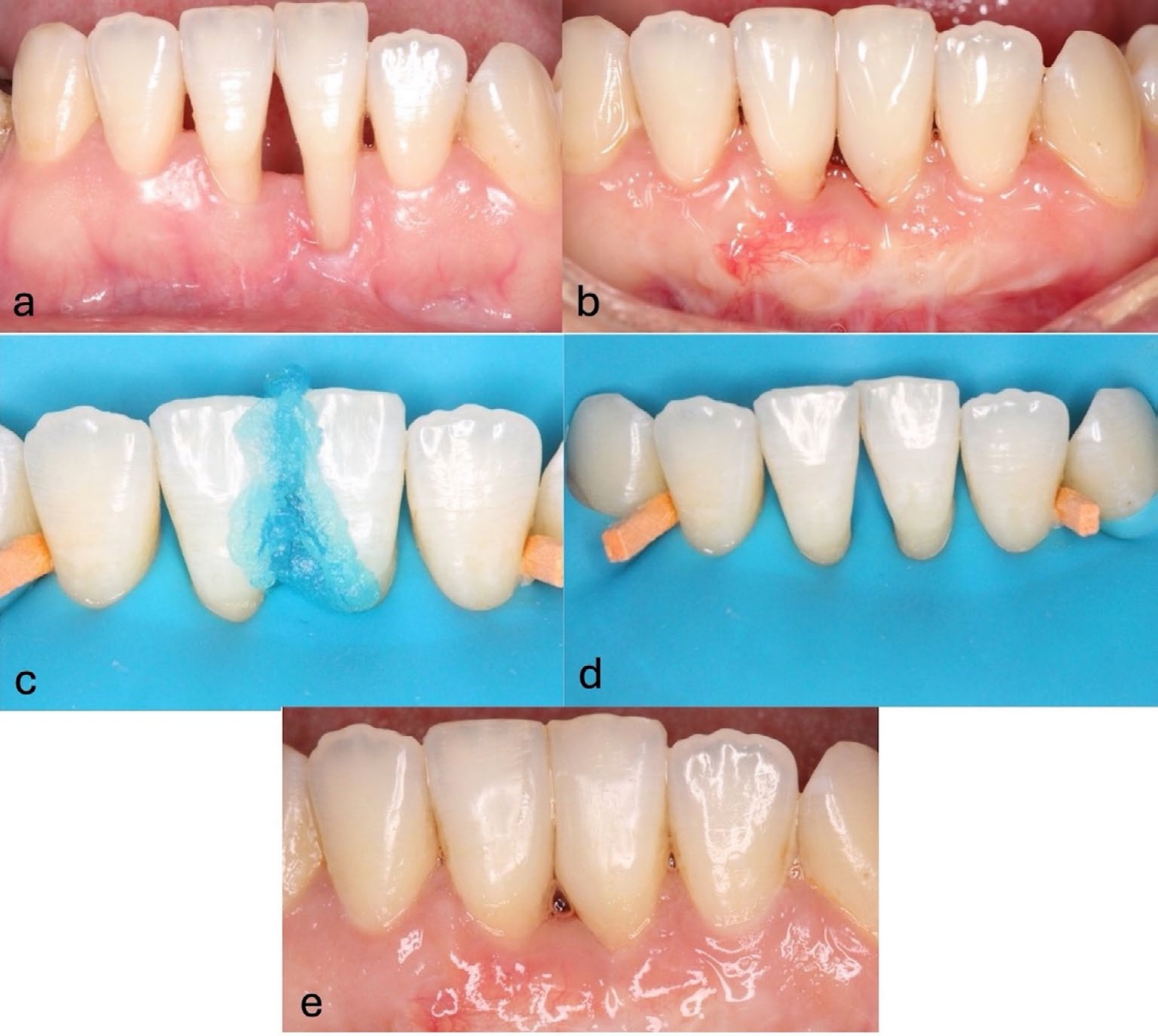
A nonsmoking, systemically healthy 66‐year‐old female. Clinical and radiographic examination revealed recession type 3 (RT3) gingival recession in her anterior mandible. (a) Clinical photo showing the recession defect at teeth #24 and #25, with visible soft tissue loss and exposure of the underlying root surfaces. (b) Six months post‐surgery, showing initial healing with partial coverage of the gingival recession. (c and d) Restorative work performed under rubber dam isolation to close the diastema between #24 and #25 using a composite restoration, highlighting the esthetic outcome and functional improvement. (e) 24‐month follow‐up, showing the long‐term stability of the restored area, with maintained gingival health and absence of recession recurrence.

The graft was gently guided under the papilla by pulling on the sutures and then secured with a simple square knot. To enhance the coronal positioning of the papilla, a vertical double‐cross mattress suture was used, suspended at the contact point, following the method described by Zuhr et al. [22] (Figures 1 and 2). This suture technique is particularly effective in promoting the coronal movement of the papilla, which is essential for achieving optimal esthetic outcomes. In cases where the interdental contact point was loose or open, the space was filled with composite resin to stabilize the area and support the graft. This additional step helps maintain the integrity of the interproximal space and ensures that the graft remains in place during the healing process (Figures 7 and 8).

### 2.4 Postoperative Care

To ensure optimal healing and minimize complications, a comprehensive postoperative care plan was implemented. Sutures were removed after 2–3 weeks (Figures 1 and 7), allowing sufficient time for initial tissue integration and healing. For the first 3 weeks, patients were advised to avoid brushing the surgical sites to prevent irritation and promote undisturbed healing. They were also instructed to avoid consuming hard foods, which could dislodge the graft or cause trauma to the healing tissues. Additionally, patients were directed to rinse once daily with a 0.12% chlorhexidine gluconate mouthwash to maintain oral hygiene and reduce the risk of infection. After this initial healing period, gentle brushing with a soft toothbrush was permitted, allowing patients to gradually resume normal oral hygiene practices. Regular follow‐up visits were scheduled biweekly for the first month and then every 3 months to monitor healing progress, address any complications, and provide ongoing support. These visits also allowed for adjustments to the postoperative care plan as needed, ensuring that patients receive personalized care tailored to their specific healing needs.

## 3 Results

This case series included five nonsmoking, systemically healthy patients who underwent the inverted T‐shape connective tissue graft (IT‐CTG) procedure for interdental papilla reconstruction. Of these, two patients presented with esthetic concerns due to interdental papilla (IP) loss, while three patients were referred primarily for root coverage in the lower incisors; in these cases, the IT‐CTG technique was also employed to enhance the soft tissue papilla profile. All patients received detailed oral hygiene instructions and completed initial periodontal therapy prior to surgery. Surgery was performed only after a full‐mouth plaque score (FMPS) < 20% and absence of bleeding on probing at the recipient site. Alternative treatments were discussed, and informed consent was obtained from all participants.

Clinical measurements—including pocket probing depths (PPD), recession heights (REC), and clinical attachment levels (CAL)—were recorded using a calibrated UNC15 periodontal probe. Embrasure fill was evaluated using Jemt's papilla index [23] (see Table 1). Quantitative results revealed that complete papilla fill (Jemt index score 2) was achieved in two sites (both RT3 cases; 40%) following adjunctive restorative procedures, while partial fill (Jemt index score 1–2, representing 50%–75% improvement) was observed in three RT2 sites (60%). The mean Jemt's papilla index improved from a baseline of 0.2 to 1.8 at the final follow‐up. Esthetic outcomes were acceptable, and morbidity was minimal across all patients.

**TABLE 1:** Provides a summary of patient characteristics, RT classification, facial recession, root coverage, Jemt's papilla index before and after treatment, specific location of treated site, and follow‐up details.

No	Age	Gender	Medical history	Smoking	Treated site location	Facial recession baseline (mm)	Root coverage achieved	RT classification	Jemt's papilla index before	Jemt's papilla index after	Follow‐up
1	33y	Male	ASA I	N	Mandibular two centrals	5 mm	Partial (2–3 mm)	RT3	0	2	6 m
2	47y	Female	ASA I	N	Mandibular two centrals	6 mm	Partial (4–5 mm)	RT3	0	2	12 m
3	43y	Female	ASA I	N	Maxillary two centrals	3 mm	Full coverage	RT2	1	2	6 m
4	53y	Male	ASA I	N	Mandibular central and lateral incisors	5 mm	Partial (2–3 mm)	RT2	0	1	3 m
5	66y	Female	ASA I	N	Maxillary two centrals	3 mm	Full coverage	RT2	0	2	6 m

Abbreviations: ASA, American society of anesthesiologist; m, months; mm, millimeter; N, no; RT, recession type; y, years.

## 4 Discussion

The IT‐CTG technique, combined with microsurgical methods, represents a significant advancement in the management of deficient interdental papillae in cases of advanced gingival recession. Our case series, which achieved complete papilla fill in 40% of sites and partial fill in 60%, demonstrates outcomes at or above the levels typically reported for traditional soft tissue grafting techniques—particularly noteworthy given the complexity of RT2 and RT3 defects addressed. This favorable outcome may be attributed to several features of the IT‐CTG approach: the T‐shaped graft configuration provides enhanced volume and stability under the papilla, the minimally invasive tunnel approach maintains blood supply, and adjunctive use of composite resin supports papilla height and tissue adaptation.

Numerous techniques have been developed to preserve papilla tissue integrity during reconstructive procedures, including the semilunar coronally advanced flap technique described by Tarnow [7]. These methods often utilize remote incisions, a concept similar to the modified tunnel approach used in this case series. The tunnel technique, which involves making incisions apically or laterally to the interproximal area for graft insertion, minimizes tissue trauma and preserves the natural vascularization of the recipient site. This approach is crucial for graft survival and integration.

Traditional papilla reconstruction strategies, including free epithelialized gingival grafts and standard connective tissue grafts, have generally demonstrated variable and often unpredictable results—particularly in cases involving advanced recession or compromised vascularity [3]. Recent literature underscores the benefits of microsurgical and tunneling techniques for enhancing root coverage and esthetic outcomes by allowing for more precise soft tissue manipulation and reduced trauma [3, 10]. However, even with these advances, reports by Han and Takei [8] as well as Froum et al. [9] indicate that the rates of complete papilla regeneration remain limited. In comparison, the outcomes in this series—achieving complete papilla fill in 40% of sites (RT3 cases), partial fill in 60% (RT2 cases), and a mean Jemt's papilla index improvement from 0.2 to 1.8—fall within the upper range of previously reported values, especially given the complexity of the treated defects [21].

Reconstructing the interdental papilla and addressing RT2 and RT3 gingival defects, which are often associated with complete papilla loss, is highly challenging. The technique described in this study utilizes a connective tissue graft in a T‐shaped configuration (IT‐CTG) to increase the thickness of the interdental papilla, encouraging papilla regeneration over time with the supplementation of restorative treatment in some cases, and providing coverage of the facial root at the same time. Despite this, achieving optimal outcomes with current methods remains difficult due to the biological challenge.

The proposed surgical technique offers several potential advantages for treating RT2 and RT3 defects with simultaneous IP reconstruction. This clinical report describes a surgical procedure that successfully achieved long‐term reformation of the IP, providing significant esthetic improvements. Since the base of the papilla is preserved, the modified tunnel technique presented is minimally invasive and maintains the blood supply to the papilla. The use of a semilunar incision or similar strategies allows for the passive displacement of interproximal soft tissue, enabling the passage of a CTG into the tunnel positioned coronal to the interproximal bone crest [9, 16, 21, 24]. While the maxillary tuberosity is often selected as a donor site in the literature due to its dense, fibrous connective tissue and greater resistance to retraction [25], our T‐shaped graft was generally harvested from the palate in all cases, providing good stability beneath the papilla and preventing twisting during suture placement. Notably, in one of the five cases in this series, the IT‐CTG technique was modified by combining a separately harvested tuberosity graft with the palatal connective tissue graft, following the “iceberg”‐CTG (iCTG) approach described by Urban et al. [26] (Figure 6). This modification may offer additional soft tissue volume in select situations but requires specialized microsurgical skills. Postoperative healing was rapid and uneventful in most patients, with wound closure typically achieved within 1 to 2 weeks. However, as with all microsurgical procedures, risks such as papilla necrosis may occur if vertical incisions are placed too close to the papilla or the interdental tissue is traumatized, emphasizing the importance of careful surgical planning and precision.

When the supracrestal thickness of the interproximal soft tissue is less than 1.5 mm, both the superficial and deeper tissue layers may be insufficient to ensure graft survival and stability. Careful management of flap tension during surgery is therefore crucial for proper tissue adaptation and to minimize excessive mechanical stress at the surgical site. According to recent literature, the presence of papillary frenal attachments with muscle fibers extending close to the interdental papilla can contribute to postoperative tension, which may impede soft tissue regeneration and clinical attachment level (CAL) gains, potentially resulting in apical migration of the repositioned tissues. In our series, to address persistent tension in such cases, an additional lower lip frenectomy was performed 2 to 6 months after the 3DT procedure [21].

Restorative treatment can also compensate for any residual papilla deficiency, as the papilla gradually “creeps” over time without showing clinical signs of inflammation. This phenomenon is supported by recent studies demonstrating that creeping attachments can occur and remain stable even after several months, contributing to improved esthetic and functional outcomes [27, 28]. However, careful selection of patients and sites is essential. This technique is not recommended for smokers or individuals with systemic risk factors associated with impaired wound healing, as smoking can negatively affect wound healing and graft success (Figure 8).

Future studies should include larger, controlled cohorts to further validate the efficacy and predictability of the IT‐CTG technique for interdental papilla reconstruction. Based on current evidence and anatomical considerations, ideal candidates are patients with diastema, large papillae, and thick gingival biotypes, as these characteristics are associated with more favorable and consistent outcomes. Research should also focus on refining case selection criteria—including quantitative assessment of papilla height limits and morphology, as described by Tarnow et al. [7]—to optimize patient‐specific treatment planning. Additionally, studies are needed to evaluate long‐term stability, patient‐centered esthetic outcomes, and the comparative effectiveness of IT‐CTG against other soft tissue grafting methods. Given the technique's dependence on surgical expertise and favorable anatomy, further innovation in minimally invasive approaches and training protocols may broaden applicability and improve reproducibility, especially for patients with thin or narrow interdental papillae or those with systemic risk factors negatively affecting wound healing.

## 5 Conclusion

In this case series, the inverted T‐shape connective tissue graft (IT‐CTG) technique achieved complete papilla fill in 40% of cases (RT3 defects) and partial fill in 60% (RT2 defects), with a mean improvement in Jemt's papilla index from 0.2 to 1.8 and minimal morbidity reported. These outcomes indicate that IT‐CTG can offer meaningful esthetic and functional benefits for interdental papilla reconstruction in carefully selected patients with advanced gingival recession, particularly where conventional techniques are less predictable. However, longer‐term and larger‐scale studies are recommended to further establish their efficacy and long‐term stability compared to established techniques.

## Author Contributions

A.E.A. led conceptualization, methodology, and project administration; contributed equally to the investigation; and served as lead author of the original draft. V.F.C. provided support in conceptualization and original‐draft writing, contributing equally to the investigation. J.L. supported conceptualization and methodology and assisted with original‐draft writing. P.D.M. and H.‐L.W. contributed to the review and editing of the manuscript. All authors reviewed and approved the final manuscript and agreed to be accountable for all aspects of the work, adhering to CRediT guidelines and established authorship practices.

## Conflicts of Interest

The authors declare no conflicts of interest.

## Supporting information


**Video S1:** A nonsmoking, systemically healthy 66‐year‐old female presented with recession type 2 (RT2) gingival recession at the maxillary anterior sites #8 and #9. Clinical and radiographic examination revealed significant gingival tissue loss and root exposure. The video demonstrates key steps of the inverted T‐shape connective tissue graft (IT‐CTG) procedure for papilla reconstruction between the central incisors. The sequence includes pre‐surgical clinical views of the recession defect, tunneling of the mesio‐buccal sulcus with a microsurgical instrument to create a recipient site while preserving vascular supply, and a vertical incision apical to the defect to facilitate graft placement. The placement and adaptation of the in situ CTG at the #8/#9 interdental papilla, as well as at site #6 to enhance esthetic and functional outcome, are shown. Immediate post‐surgical footage displays tension‐free closure with sutures securing the grafts. The video concludes with 6‐month follow‐up, demonstrating complete root coverage, successful graft integration, and healthy maintained gingiva at the treated sites.

## Data Availability

The data that support the findings of this study are available on request from the corresponding author. The data are not publicly available due to privacy or ethical restrictions.
